# The effect of weight loss intervention programme on health-related quality of life among low income overweight and obese housewives in the MyBFF@home study

**DOI:** 10.1186/s12905-018-0591-3

**Published:** 2018-07-19

**Authors:** Rashidah Ambak, Noor Safiza Mohamad Nor, Norhanizam Puteh, Azmi Mohd Tamil, Mohd Azahadi Omar, Suzana Shahar, Noor Ani Ahmad, Tahir Aris

**Affiliations:** 1 Institute for Public Health, National Institutes of Health, Ministry of Health Malaysia, Kuala Lumpur, Malaysia; 2 Department of Community Health, Faculty of Medicine, Universiti Kebangsaan Malaysia Medical Centre, Kuala Lumpur, Malaysia; 3 Department of Nutrition and Dietetics, Faculty of Allied Health Sciences, Universiti Kebangsaan Malaysia, Kuala Lumpur, Malaysia

**Keywords:** Health related quality of life (HRQOL), Obesity and weight loss quality of life (OWLQOL), Obesity, Housewives

## Abstract

**Background:**

Obesity is an emerging global public health concern as it is related to chronic diseases and its impact to health related quality of life. The aim of this study was to assess the effect of weight reduction on health related quality of life (HRQOL) among obese and overweight housewives.

**Methods:**

Data on 123 obese and overweight housewives in the intervention group from the MyBFF@home study were utilised. A validated Malaysian Malay version of Obesity Weight Loss Quality of Life (OWLQOL) questionnaire was administered at baseline and 6 months after intervention. Descriptive analysis, univariate analysis, paired t-test and multiple logistic regression were performed using SPSS Version 22.

**Results:**

Mean body mass index (BMI) was 31.5 kg/m^2^ (SD:4.13), with 51 participants classified as overweight (41.5%) while 72 were obese (58.5%). About 72% of the housewives experienced weight reduction (62% reduced weight less than 5% and 11% reduced weight more than 5% of their baseline weight). There was a significant improvement in HRQOL with a pre-intervention total mean score of 59.82 (SD: 26.60) and post-intervention of 66.13 (SD: 22.82), *p*-value < 0.001. By domain, the highest post intervention mean score was self-image 71.46 (SD: 22.85), followed by social stigma 68.77 (SD: 28.70), physical 61.83 (SD: 24.25) and trying to lose weight 61.24 (SD: 27.32). There was no significant association between weight reduction and HRQOL improvement.

**Conclusion:**

Weight loss intervention programme utilizing behavioural modification has led to a significant improvement in HRQOL among overweight and obese housewives.

## Background

Obesity is becoming of epidemic proportions worldwide. It has been approximated that there will be an increase in the world overweight or obese adult’s populations from 33% in 2005 to 58% by 2030 if the current trends persist [1]. This phenomenon of increasing obesity caused a global burden due to increase in chronic diseases and disability as obesity leads to several physical, psychological and social problem [2]. Physically, obesity is associated with hypertension, hyperlipidaemia, type 2 diabetes, coronary heart disease, arthritis and certain type of cancer [1, 2]. Psychologically, obesity is associated with lower self-esteem, negative self-evaluation and decreased self-image, and socially, an obese person often faces discrimination and prejudice, thereby causes negative economic and social consequences [2, 3].

In general, the above consequences often impaired the health related quality of Life (HRQOL) of the obese individual. HRQOL is a self-reported outcome from the affected individuals concerning their life, health condition and treatment. This concept is correlated to an individual’s feeling of well-being and perceptions of physical, emotional and social functioning which manifest in the personal reaction and evaluations towards their health condition [4]. The most common and immediate consequence from obesity is the negative impact on the HRQOL particularly among women [5]. A few studies indicated strong relationships between obesity and quality of life, where the quality of life decreases as weight increases [6–8].

A study among non-pregnant productive age women in Turkey on the relationships between obesity and HRQOL using World Health Organization Quality of Life Questionnaire abbreviated version (WHOQOL-BREF) showed that overweight and obese prevalence were increasing with aging, lower education level and low socio-economic status [9]. Among them, 14.7% reported suffering from chronic illness were overweight and obese. After adjusting for age, level of education and co-morbid illness, the obese (BMI ≥ 30 kg/m^2^) women had a significant lower HRQOL scores on all domains except environment. This study suggested that overweight and obese not only increase morbidity and mortality, but it could also lead to negative contribution to individual HRQOL [9].

There were complex relationships between obesity and common mental health disorder. According to the National Obesity Observatory 2011, an overview on the current association of obesity and mental health among adults and children in United Kingdom, there were bi-directional association between obesity and common mental disorder such as depression and anxiety [10]. It also discusses the issue related the health inequalities and the implication of psychological distress caused by weight related stigma and discrimination [10]. However, most theories emphasise that obesity causes increased medical illness and restriction of mobility which have direct impact on psychological well-being, whereby leading to low self-esteem, eating disorder, distorted body image, anxiety and depression [11]. Unfavourable psychological factors, lower self-rating of health and worse health related behaviour can be found in overweight and obese individual [12, 13]. Being obese reduces their self-esteem and the effect on their social life leaving them isolated and vulnerable [14].

A study regarding self-perception and quality of life among overweight and obese rural housewives in Kelantan, Malaysia found they have dangerous perceptions of obesity where more than 55% perceived that obesity symbolised happiness and conversely, equated to unhappiness [15]. Even though most of the participants were aware of their body weight and had intentions to lose weight, they still reported themselves as healthy or very healthy. This suggests that public health approach for rural housewives needs to be tailored to health-related consequences of obesity/overweight.

There are many studies documenting strong association between obesity with morbidity and mortality, however a few studies regarding the impact of being overweight or obese on HRQOL exist especially in Malaysia. As Malaysia is the country with the highest obese population among the Southeast Asia countries [16] and housewives have higher BMI compared to other job categories [17], therefore the impact of obesity on HRQOL especially among obese and overweight housewives is essential to be explored. A good HRQOL will improve the quality of life of the individuals, families, communities, and health status of the population. Hence it will prevent chronic diseases and mental disorders such as low self-esteem, anxiety and depression. The aim of this study is to assess the effect weight reduction on the HRQOL among the obese and overweight housewives who participated in the My Body is Fit and Fabulous at Home (MyBFF@home) study.

## Methods

MyBFF@home was a quasi-experimental study conducted within the community settings in the Federal Territory of Kuala Lumpur, Klang Valley. In the MyBFF@home, 328 housewives living in the low cost flats (People’s Home/Housing project) were recruited and divided into the control group (*n* = 159) and intervention group (*n* = 169). Screening for the housewives was conducted by the health clinics staffs with support from the respective community representatives. Subsequently, prior to the baseline assessments, the researchers set appointments with the housewives to explain the study and to get their written consents. Housewives included in this study were defined as married, single or widowed women, aged between 18 and 59 years old, without a job or with a part-time job (less than 6 h work per day), and overweight or obese housewives (BMI between 25.0–39.9 kg/m^2^)^21^. The exclusion criteria were housewives who were morbidly obese (BMI ≥ 40 kg/m^2^), had chronic diseases such as diabetes, heart disease, renal disease, those with moderate or severe hypertension and require medication, those with limitation for physical activity (physical disability, bed ridden), currently participating in the weight management programme, pregnant and those who were unable to communicate either in Malay or English. Weight loss was targeted of at least 5% of the baseline weight [18]. The details of the MyBFF@home were described elsewhere [19] and in the present report (Introduction of the MyBFF@home). For the purpose of this paper, data of participants in the intervention group at baseline (pre-intervention) and after 6 months (post-intervention) were explored. A total of 169 housewives in the intervention group answered the self-administered HRQOL questionnaire. Total number of participants who completed 6 months intervention was 123, with the attrition rate of 26%.

Prior to the intervention, the health team from Department of Health Federal Territory of Kuala Lumpur performed health screening to confirm the health status of the participants. Measurements on height, weight and BMI were done throughout the intervention period, and questionnaires were administered to gather data related to socio-demographic, socio-economic and health status. Since this study was conducted among the low income group, the mean of monthly household income of of RM1800 from Malaysian Statistic Department was used to categorise the participants into two income groups [20].

### HRQOL measurement using the OWLQOL – Malaysian Malay version

The HRQOL among the participants was measured using a validated Malaysian-Malay Obesity and Weight-Loss Quality of Life (OWLQOL) questionnaire [21]. The OWLQOL is a self-administered questionnaire and evaluates the feelings of participants about obesity and their effort to lose weight [21]. It constituted of 4 domains with 17 items, which were self-image (7 items with α = 0.907), social stigma (2 items with α = 0.851), trying to lose weight (3 items with α = 0.767) and physical (5 items with α = 0.872). Responses indicated were seven–point scale that ranges from 0 (‘not at all’) to 6 (‘a very great deal’) [22]. Each item scale was reversed before the total score was calculated. Subsequently, the total score was transformed to 0 to 100 scales, with higher scores representing better obesity-specific quality of life [22]. The questionnaire was answered by participants at baseline (pre-intervention) and 6-month follow up (post-intervention). In the MyBFF@home study, the questions have been translated to the Malaysian-Malay version before piloted among 28 overweight and obese female health staffs. The participants took between 10 to 15 min to complete the questionnaire and the Cronbach Alpha (α) was 0.953 [23].

### Body weight, height and body mass index (BMI)

Body weight was measured with a digital scale (Tanita HD319, Japan) in kilogram (kg), to the nearest 0.1 kg. Participants were measured in light clothing and no shoes. Body height was measured from head to toe in an upright standing position with five points of his body touching the wall with a SECA Bodymeter in cm, to the nearest 0.1 cm (cm). Both weight and height were measured twice, and the average value of the measurements was computed. BMI was calculated by dividing the measured body weight (kg) by the squared measured body height (m^2^). The WHO 1998 classification^21^ was referred with regards to the BMI classification (normal: 18.5–24.9 kg/m^2^, overweight: 25.0–29.9 kg/m^2^, obese type I: 30.0–34.9 kg/m^2^ and obese type II: 35.0–39.9 kg/m^2^).

### Statistical analysis

Statistical analysis was conducted using Statistical Package for Social Science (SPSS version 22.0). Normality test was done and the data showed a normally distributed except for the difference of HRQOL. Descriptive analysis of the participants was calculated as frequency (n) and percentage (%) or mean (SD). For univariable analysis, paired t-test was used to test for mean differences of HRQOL and weight reduction, pre and post intervention. Chi square analysis was used to determine the association between the categorical variables of age group, ethnicity, marital status, education level, household income group and weight improvement group with improvement in HRQOL. Further analysis of the data was done using multiple logistic regressions. A *p*-value of < 0.05 was considered as statistically significant.

## Results

Table 1 shows the subject characteristics for participant who completed the 6 months intervention programme. At baseline, there were 167 participants involved in this programme. After 6 months, 123 (73%) participants were still involved. A series of independent sample t-test and Chi square test found no significant differences on any of the demographic variables at baseline for participants who withdrawn from the programme compared to those who completed the intervention. Majority of the participants were Malay (88.6%) and from 40 to 49 year old age group (42.2%) with a mean age of 42.8 years (SD: 8.0). Most of them were married (89.4%) and studied until secondary school of 12 to 18 years old (82.9%). The mean household income was RM 1794 (low socio-economic status), with a minimum and maximum income of RM800 and RM4,000, respectively).

**Table 1:** Socio-demographic and socio-economic characteristics of housewives who completed the 6 months programme

Variable	Mean (SD)	N	(%)
Age Group (Years)	42.81 (8.00)		
18–29		6	4.9
30–39		37	30.1
40–49		52	42.2
50–59		28	22.8
Ethnic Group			
Malay		109	88.6
Chinese		3	2.4
Indian		8	6.6
Others		3	2.4
Marital Status			
Married		110	89.4
Widow/Divorcee		13	9.6
Education Level			
Primary		21	16.9
Secondary		103	83.1
Monthly Household Income (RM)			
≤ RM 1800	RM 1794.00 (825.74)	71	57.7
> RM 1800		52	42.3

*SD* Standard deviation

Baseline mean weight was 76.0 kg (SD:11.24) with the maximum weight recorded as 111 kg. Mean body mass index (BMI) was 31.5 kg/m^2^ (SD:4.13), with 51 participants classified as overweight (41.5%) while 72 were obese (58.5%). About 72% of the housewives experienced weight reduction There was a significant weight reduction of 1.25 kg (SD: 2.41), *p* <  0.001.

### Quality of life changes

Out of 123 participants, 77 (62.6%) participants showed improvements in the HRQOL, while 46 (37.4%) participants had no improvement. The mean OWLQOL score at baseline was 59.83 (SD:26.60) compared to 66.14 (SD:22.83) after 6 months intervention. There was a significant difference between the mean of the OWLQOL total score at baseline compared to 6 months intervention which signified a significant improvement in HRQOL, with a mean difference of 6.32 (SD:19.28) and *p* <  0.001. Total score in all domains increased significantly after the 6 months intervention compared to baseline (Table 2). The physical domain showed the highest significant increase in quality of life (*p* <  0.001).

**Table 2:** Changes in HRQOL Domain

Quality of life domains	Baseline score mean (SD)	6 months post intervention mean (SD)	*p*-value*
Self-image	64.81 (27.84)	71.46 (22.85)	0.001
Social stigma	63.96 (33.29)	68.77 (28.70)	0.048
Trying to lose weight	55.41 (29.66)	61.24 (27.32)	0.008
Physical	53.97 (26.80)	61.83 (24.25)	< 0.001

*Paired t-test with *p*-value significant at < 0.05

In Table 3, the HRQOL was assessed based on condition-specific questions. The lowest mean score at baseline was for the question ‘I have to pay close attention to personal hygiene’ 2.78 (SD: 2.00), ‘I worry about the future because of my body weight’ 2.83 (SD: 2.17) and ‘I am afraid that I will gain back any weight lost’ 2.99 (SD: 2.08). There were significant differences in HRQOL in 11 out of 17 condition-specific HRQOL, between pre and post 6-month intervention. The highest mean difference in improvement was ‘I worry about the future because of my body weight’ (mean difference = 0.71 (SD: 2.05), *p* <  0.001).

**Table 3:** Changes of Obesity and Weight Loss Quality of Life (OWLQOL) based on condition

Condition (scoring: minimum = 0, maximum = 6)	Baseline	Post 6 months intervention	*p*-value*
	Mean (SD)	Mean (SD)	
Because of my weight, I try to wear clothes that hide my shape	3.46 (1.85)	3.73 (1.85)	0.103
I feel frustrated that I have less energy because my weight	3.50 (1.98)	3.84 (1.68)	0.046
I feel guilty when I eat because of my weight	3.43 (1.94)	3.71 (1.80)	0.143
I am bothered about what people say about my weight	3.60 (2.10)	4.01 (1.89)	0.019
Because of my weight, I try to avoid having my photograph taken	4.13 (2.07)	4.48 (1.84)	0.024
Because of my weight, I have to pay close attention to personal hygiene	2.78 (2.00)	3.00 (1.95)	0.245
My weight prevents me from doing what I want to do	3.88 (2.06)	4.42 (1.86)	0.002
I worry about the physical stress that my weight put on my body	3.16 (2.05)	3.74 (1.96)	0.001
I feel frustrated that I am not able to eat what others do because of my weight	4.28 (2.00)	4.37 (1.77)	0.613
I feel depressed because of my weight	4.31 (2.03)	4.76 (1.68)	0.007
I feel ugly because of my weight	3.80 (2.11)	4.44 (1.90)	< 0.001
I worry about the future because of my weight	2.83 (2.17)	3.54 (2.09)	< 0.001
I envy people who are thin	3.02 (2.22)	3.49 (2.08)	0.003
I feel people stare at me because of my weight	4.08 (2.12)	4.24 (1.84)	0.218
I have difficulty accepting my body because of my weight	3.83 (2.07)	4.26 (1.84)	0.020
I am afraid that I will gain back any weight that I lose	2.99 (2.08)	3.08 (2.07)	0.642
I get discouraged when I try to lose weight	3.95 (2.12)	4.44 (1.94)	0.008

Higher scores indicate less affected by the condition and better HRQOL

*Paired t-test with *p*-value significant at <0.05

### HRQOL with weight reduction

**Fig. 1:**
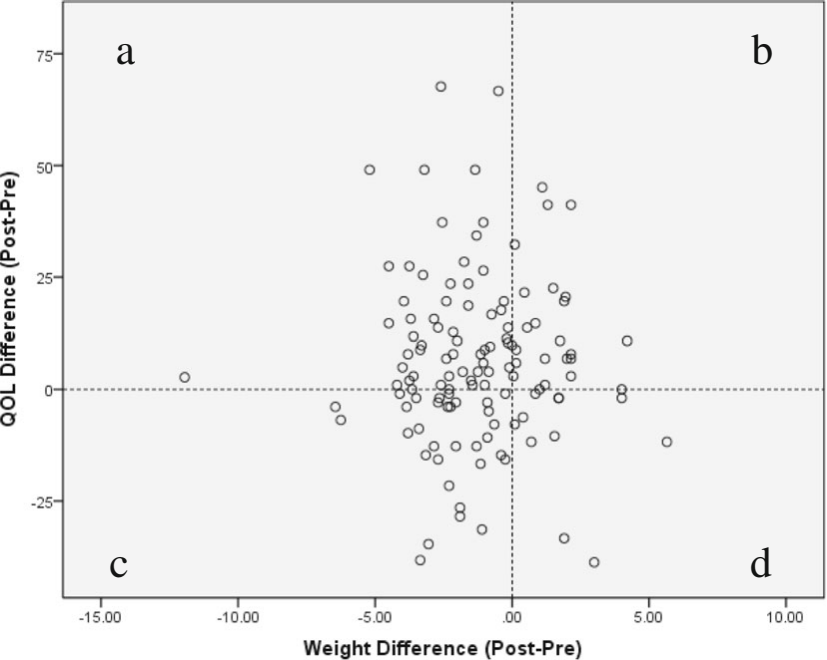
Relationship between differences in HRQOL with differences in weight reduction. **a** Weight reduced and HRQOL improved (44.5%) **b** Weight increased and HRQOL improved (18.1%) **c** Weight reduced and HRQOL reduced (28.2%) **d** Weight increased and HRQOL reduced (9.2%)

About 72% of the housewives experienced weight reduction (62% reduced weight less than 5% of their baseline weight and only 11% reduced weight more than 5% of their baseline weight). Figure 1 showed relationship between weight differences post intervention and changes in HRQOL. About 44.5% of the participants experienced weight reduction with improvement in HRQOL, while 18.1% reduced weight but reported no improvement in HRQOL. Further analysis showed no significant relationship between improvement in HRQOL with amount of weight reduction. Chi square tests and multiple logistic regressions showed no significant association between weight difference (lose weight or not) and BMI group (overweight and obesity) with improvements in HRQOL.

## Discussion

Findings showed that involvement in weight loss intervention focusing on behaviour modification in 6 months duration resulted in the improvement of the HRQOL. This was similar to an intervention study on HRQOL following a weight loss intervention programme among overweight and obese adults in the United States of America where there was a peak of improvement in HRQOL at the end of 6 months intervention [24]. Kaukua et al. (2003) also reported similar findings among obese adults following weight lost intervention [25]. Similar findings were found in both interventions which focused on behaviour modifications, and thus improving the HRQOL.

The highest post intervention domain mean score was self-image, followed by social, physical and trying to lose weight. The lowest condition-specific mean score at baseline of ‘I have to pay lose intention to personal hygiene’ showed that overweight and obese housewives were worried about their personal hygiene due to excessive body weight. Highest mean difference in improvement on the condition-specific was ‘I worry about the future because of my body weight’ showed that they become less worried on the impact of obesity in the future since they were involved in the weight loss intervention programme, where they felt happy, engaged with healthy lifestyle. This finding was similar in a weight loss intervention study using dietary and physical activity in Taiwan, where the 4 domains of HRQOL showed improvements after the intervention [26].

About 72% of the participants experienced weight reduction, but the mean reduction was less than 5% of the baseline weight. There was no significant association between improvements of HRQOL with weight reduction. Those who achieved weight reduction may or may not have improved HRQOL. Those who did not reduce weight also may or may not have improved HRQOL. The improvement of HRQOL was also not associated with age group, ethnicity, marital status, educational level and income. The lack of significant association between the amounts of weight reduction with the increased in HRQOL has been reported in few studies. Kolotkin et al. (2001) reported only 14% of changes in HRQOL scales could be explained by weight loss [27]. Similarly, another study reported that only 2 out of 7 quality of life measures were different among individuals who lost more than 5% of their weight compared to those had stable weight [26]. Frontaine et al. (2001) also reported no difference in improvement of HRQOL among participants who maintained their weight loss or regained their weight, if they continue the intervention up to 2 years [28]. There is a need to develop a better understanding of what would lead to improvement in the HRQOL among overweight and obese adults. It is possible that satisfaction on behaviour modification such as exercising in group, together with healthy diet can explain the improvements in the HRQOL. It is also possible that social interactions while getting involved in the program, weight loss intervention support by the health staffs and involvement in the community intervention were responsible for some of the improvements in the HRQOL. This aspect could be explored further in future research.

The strength of this study is using the OWLQOL questionnaire, which is specific to evaluate the feelings of participants regarding obesity and the effort in trying to lose weight. It measures a person’s global evaluation of position in life related to weight, weight loss and weight loss treatment. The OWLQOL also involved a multicultural item generation in development of obesity-specific measures which measure the specific and concurrent inclusion of items from multiple cultures. This is important since attitudes towards obesity and overweight have differing relevance, importance and sensitivity across different culture [26]. Other HRQOL questionnaires such as World Health Organization Quality of Life (WHOLQOL-BREF), Medical Outcome Study (MOS), Short Form-36 (SF-36) used a generic measure of HRQOL which do not specifically evaluate position as an obese person and their effort to lose weight. The questionnaire might only suitable for some cultures [29]. The present report only reported assessment of the HRQOL was at baseline and 6 months intervention. Further analysis after the post intervention phase is needed to evaluate any significant correlation between improvements in the HRQOL with weight reduction. The OWLQOL is a self-reported questionnaire, therefore there will be some information bias which may influence the impact of weight reduction towards the HRQOL among the participants.

## Conclusions

The findings showed that overweight and obese housewives experienced significant improvements in HRQOL after involved in a 6 months community-based weight loss intervention programme either with or without weight loss. Therefore, overweight and obese housewives are recommended to join a community-based weight loss intervention programme to improve their quality of life. Further study is necessary to determine the contributing factor that can improve the quality of life among the overweight and obese housewives in the Klang Valley urban area.

## Abbreviations

BMI: Body Mass Index
HRQOL: Health related quality of life
MOS: Medical Outcome Study
MyBFF@home: My Body is Fit and Fabulous at home
SF-36: Short Form-36
WHOQOL-BREF: World Health Organization Quality of Life Questionnaire abbreviated version
